# Modeling of Rate-Dependent Hysteresis Using a GPO-Based Adaptive Filter

**DOI:** 10.3390/s16020205

**Published:** 2016-02-06

**Authors:** Zhen Zhang, Yaopeng Ma

**Affiliations:** School of Automation Sciences and Electrical Engineering, Beihang University, XueYuan Road NO.37, Haidian District, Beijing 100191, China

**Keywords:** hysteresis, rate-dependent, adaptive filter, LMS, modeling

## Abstract

A novel generalized play operator-based (GPO-based) nonlinear adaptive filter is proposed to model rate-dependent hysteresis nonlinearity for smart actuators. In the proposed filter, the input signal vector consists of the output of a tapped delay line. GPOs with various thresholds are used to construct a nonlinear network and connected with the input signals. The output signal of the filter is composed of a linear combination of signals from the output of GPOs. The least-mean-square (LMS) algorithm is used to adjust the weights of the nonlinear filter. The modeling results of four adaptive filter methods are compared: GPO-based adaptive filter, Volterra filter, backlash filter and linear adaptive filter. Moreover, a phenomenological operator-based model, the rate-dependent generalized Prandtl-Ishlinskii (RDGPI) model, is compared to the proposed adaptive filter. The various rate-dependent modeling methods are applied to model the rate-dependent hysteresis of a giant magnetostrictive actuator (GMA). It is shown from the modeling results that the GPO-based adaptive filter can describe the rate-dependent hysteresis nonlinear of the GMA more accurately and effectively.

## 1. Introduction

Smart actuators, such as piezoelectric actuators (PEAs), giant magnetostrictive actuators (GMAs) and shape memory alloys (SMAs), have great potential in micro-positioning and micro-vibration control [1,2]. Owing to some of the magneto-electro-thermo-elastic coupling effects in smart materials, smart actuators exhibit dynamic hysteresis nonlinearity, making their effective use quite challenging.

Hysteresis modeling methods can be roughly divided into physical-based models, such as the Jiles-Atherton model for ferromagnetic materials [3], the free energy model for ferroelectric materials [4], the domain wall model for piezoelectric materials [5] and phenomenological models, including the Preisach model [6], the Krasnoselskii-Pokrovskii (KP) model [7], the Prandtl-Ishlinskii (PI) model [8,9] and the generalized Prandtl-Ishlinskii (GPI) model [10]. It should be mentioned that the classical phenomenological operator-based models describe only rate-independent hysteresis behavior. Some works have focused on rate-dependent hysteresis modeling of smart actuators. A basic idea in rate-dependent hysteresis modeling is to extend the static parameters in models to rate-dependent ones, accounting for the dependence of the weighting function on the input signal rate [11,12] or on the input signal frequency [13,14] in the Preisach model, a rate-dependent weighting function in the modified Prandtl-Ishlinskii (MPI) model [15] and rate-dependent thresholds in the GPI model [16], for example. Another idea in rate-dependent modeling is to couple the static hysteresis model to equations describing the origins of the rate-dependent behaviors. Tan presented a dynamic hysteresis model for magnetostrictive actuators by coupling a Preisach operator to an ordinary differential equation [17]. Based on equivalent energy dissipation, a rate-dependent hysteresis model for GMA was proposed by combining the MPI model with a second-order ordinary differential equation in a cascaded structure [18]. Some intelligent computation methods have been used to model rate-dependent hysteresis behavior, including neural networks [19,20], fuzzy tree [21] and support vector machine [22].

**Figure 1:**
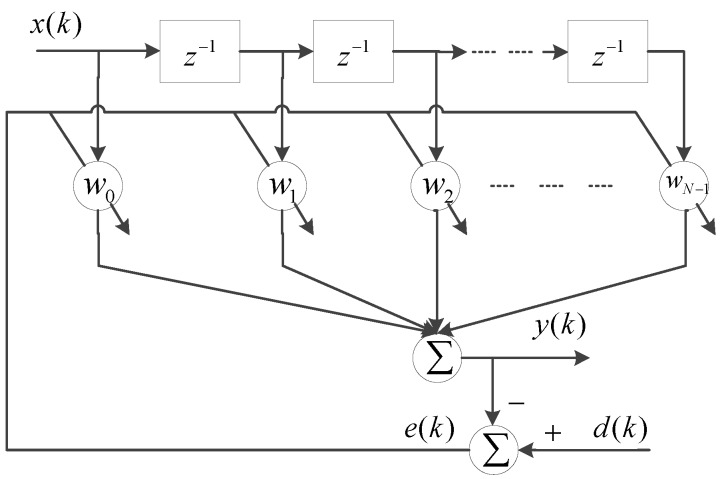
The delayed adaptive transversal filter.

In practical engineering, a nonlinear plant to be controlled may be unknown and possibly time-variable. Adaptive modeling uses adaptive filters to model a nonlinear plant. A delayed adaptive filter, shown in Figure 1, has been widely used for its simple structure and ease of implementation [23,24]. However, many experimental results show that the linear delay adaptive filter does not fit hysteresis characteristics well [25]. Using Volterra series filters as nonlinear filters is another possible choice [26]. A Volterra functional approach was presented to characterize nonlinear dynamical hysteresis based on an extension that overcame the single-valued limitation of the Volterra expansion [27]. A backlash-operator-based adaptive filter was proposed for piezoelectric actuators by replacing the delay operators in the delayed adaptive transversal filter with backlash operators [25]. This adaptive filter constitutes a Prandtl-Ishlinskii model with a substantial adaptive weight vector.

**Figure 2:**
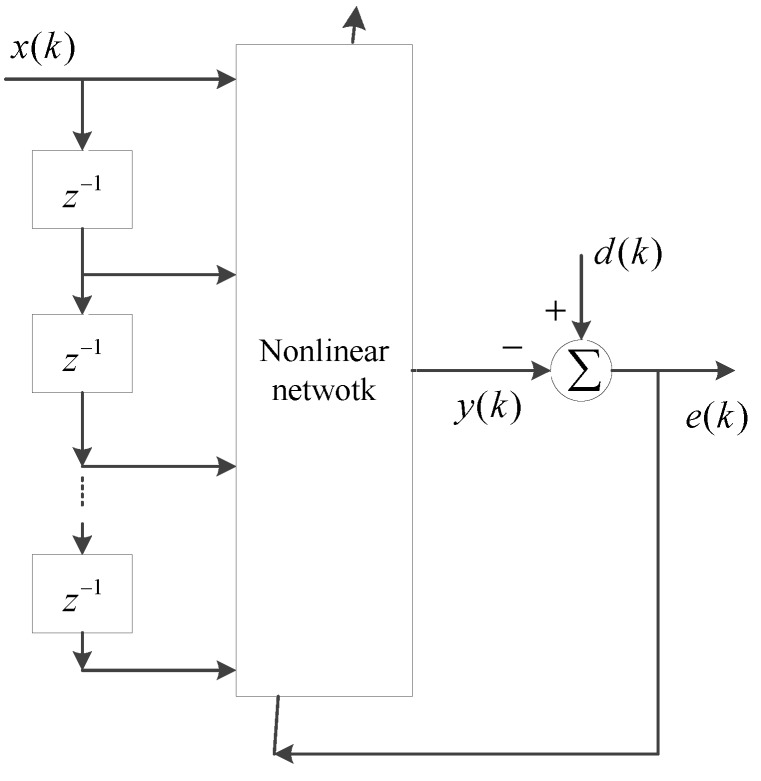
The structure of the general nonlinear filter.

The motivation for this study is to use a nonlinear adaptive filtering structure combined with hysteresis elements to better model rate-dependent hysteretic nonlinear phenomena in smart structures. A novel nonlinear adaptive filter using the general nonlinear filter structure, depicted in Figure 2, is presented for modeling rate-dependent hysteresis. The input signal vector consists of the output of a tapped-delay line with a single input signal, and generalized play operators (GPOs) with various thresholds are used to construct a single-layer nonlinear network. The output signal is composed of a linear combination of signals from the output of the GPOs. Because of the hysteresis characteristics of GPOs, a GPO-based adaptive filter can describe the rate-dependent hysteresis nonlinearity with asymmetric and saturation properties. The identification method for the parameters in the GPOs is given based on the analysis of the nonlinear filter system. In order to show the validity of the proposed adaptive nonlinear filter, four adaptive filter modeling methods are compared: GPO-based filter, backlash filter [25], second-order series Volterra filter and a linear adaptive filter. Learning algorithms are key in the performances of an adaptive filter. The LMS algorithm is widely used as a weight vector learning algorithm owing to its computational simplicity. Some variable step-size LMS algorithms have been proposed to enhance the performance of adaptive filters [28,29]. Some novel LMS algorithms were also proposed to improve the convergence and modeling errors of the Volterra filter [30,31]. In this study, the standard LMS algorithm is used in modeling experiments because the purpose of the experiments is to show the validation of the proposed modeling method by comparison of various adaptive filter modeling methods. A GMA system with strong rate-dependent hysteresis effects is used as the model plant, and various kinds of speed input signals are employed to actuate the GMA system in order to test the rate-dependent modeling capability of the proposed method.

This paper is organized as follows: In Section 2, the basic theory for GPO and GPI is given. In Section 3, the nonlinear adaptive filter is proposed for rate-dependent hysteresis modeling, and the LMS-based algorithm for weight vector adaptive learning and identification of GPO parameters in the GPOs is given. In Section 4, the proposed GPO-based adaptive filter is used to model the GMA system, and comparisons of four adaptive filter modeling methods are given. Section 5 provides conclusions.

## 2. GPO and GPI Model

### 2.1. Play Operator

**Figure 3:**
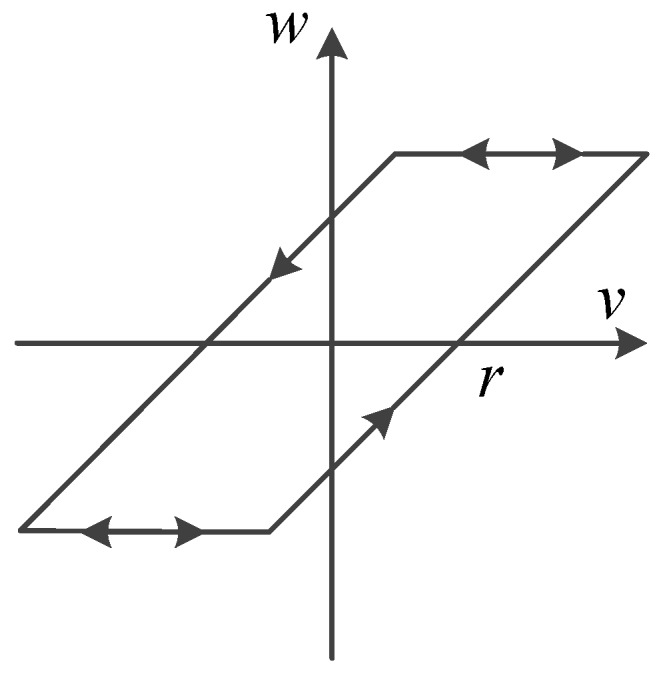
Characteristics of the play operator.

The play operator, shown in Figure 3, is the elementary hysteretic kernel in the PI hysteresis model and is a rate-independent and continuous hysteresis operator. Analytically, let $C_{m}\left[0,t_{E}\right]$ represent the space of piecewise monotone continuous functions. For any input $v\left(t\right)\in C_{m}\left[0,t_{E}\right]$, let $0=t_{0}<t_{1}<t_{2}<\cdots <t_{N}=t_{E}$ be a partition of $\left[0,t_{E}\right]$, such that the function *v* is monotone on each of the sub-intervals $\left[t_{i},t_{i+1}\right]$. Then, the output of the play operator is defined by:

(1) $$\begin{array}{ccc} F_{r}\left[v\right]\left(0\right) & = & f_{r}\left(v\left(0\right),0\right)=w\left(0\right) \end{array}$$

(2) $$\begin{array}{ccc} F_{r}\left[v\right]\left(t\right) & = & f_{r}\left(v\left(t\right),F_{r}\left[v\right]\left(t_{i}\right)\right) \end{array}$$

for $t_{i}<t\leq t_{i+1}$ and 0≤i≤N−1, where $f_{r}\left(v,w\right)=\max\left(v-r,\min\left(v+r,w\right)\right)$.

### 2.2. Generalized Play Operator

**Figure 4:**
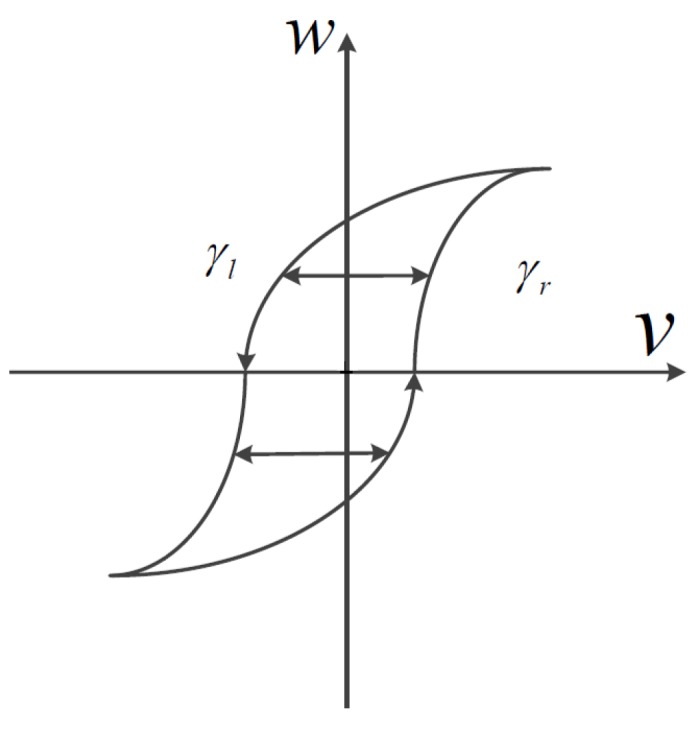
Input-output relationship for a generalized play operator.

The classic Prandtl-Ishlinskii model is limited to symmetric hysteresis loops owing to the symmetric nature of the play operator, which is the main drawback of the PI model, because it is too restrictive for real complex hysteretic nonlinearities. Therefore, a generalized play operator is given as in Figure 4 to overcome this restriction, where an increase in input *v* causes the output *w* to increase along the curve $\gamma_{r}$ or a decrease in input *v* causes the output *w* to decrease along the curve $\gamma_{l}$, with continuous non-decreasing functions $\gamma_{l}>\gamma_{r}$ named envelop functions. Analytically, for any input $v\left(t\right)\in C_{m}\left[0,t_{E}\right]$, the output of the generalized play operator is defined by:

(3) $$F_{lr}^{\gamma }\left[v\right]\left(0\right)=f_{lr}^{\gamma }\left(v\left(0\right),0\right)=w\left(0\right)$$

(4) $$F_{lr}^{\gamma }\left[v\right]\left(t\right)=f_{lr}^{\gamma }\left(v\left(t\right),F_{lr}^{\gamma }\left[v\right]\left(t_{i}\right)\right)$$

for $t_{i}<t\leq t_{i+1}$ and 0≤i≤N−1, where $f_{lr}^{\gamma }\left(v,w\right)=\max\left(\gamma_{r}\left(v\right)-r,\min\left(\gamma_{l}+r,w\right)\right)$.

The envelop functions $\gamma_{l}$ and $\gamma_{r}$ can be chosen as follows:

(5) $$\begin{array}{ccc} \gamma_{l} & = & a_{0}\tanh\left(a_{1}v+a_{2}\right)+a_{3} \end{array}$$

(6) $$\begin{array}{ccc} \gamma_{r} & = & a_{4}\tanh\left(a_{5}v+a_{6}\right)+a_{7} \end{array}$$

where $a_{j},\;j=0,\cdots ,7$ can be identified from experimental data. For a given input v(t)∈C[0,T], w∈R and $w\left(0\right)=F_{lr}^{\gamma }\left(v\left(0\right),0\right)$, the maximum and minimum values of the generalized play operator are determined by the envelope functions $\gamma_{r}$ and $\gamma_{l}$ as follows:

(7) $$\begin{array}{ccc} \max_{t\in [0,T]}F_{lr}^{\gamma }\left[v\right]\left(t\right) & = & f_{lr}^{\gamma }\left(\max_{t\in [0,T]}\gamma_{r}\left(v\left(t\right)\right),w\left(0\right)\right) \end{array}$$

(8) $$\begin{array}{ccc} \min_{t\in [0,T]}F_{lr}^{\gamma }\left[v\right]\left(t\right) & = & f_{lr}^{\gamma }\left(\min_{t\in [0,T]}\gamma_{l}\left(v\left(t\right)\right),w\left(0\right)\right) \end{array}$$

### 2.3. GPI Model

The threshold-discrete GPI model is formulated through using GPO as:

(9) $$y\left(t\right)=\sum_{i=0}^{N}w_{r_{i}}F_{r_{i}}^{\gamma }\left[v\right]\left(t\right)$$

where thresholds $r_{i}$ can be chosen to be equal intervals:

(10) $$r_{i}=\frac{i}{N+1}\max\{\gamma_{r}{(∥v∥}_{\infty }),\gamma_{l}{(∥v∥}_{\infty })\},\;for\;i=0,1,\cdots ,N$$

The weights $w_{r_{i}},\;i=0,1,\cdots ,N$ and parameters $a_{j},\;j=0,1,2,\cdots ,7$ of the envelop functions can be identified through minimization of the error sum-squared function:

(11) $$J=\sum_{l=0}^{n}{\left(y_{\gamma }\left(l\right)-y_{m}\left(l\right)\right)}^{2}$$

where $y_{\gamma }\left(l\right)$ is the model response and $y_{m}\left(l\right)$ is the measured experimental data; the index l(l=0,…,n) refers to the number of the data points considered to compute the error function. It should be noted that the identification process is iterative, as the envelop functions are initially unknown. The detailed parameter identification method can be found in [10].

## 3. GPO-Based Adaptive Filter for Rate-Dependent Hysteresis Modeling

In this section, we describe the modeling method for the rate-dependent hysteretic system using the GPO-based nonlinear adaptive filter. The structure of the GPO-based nonlinear filter is first proposed, and the parameter-identification method for the GPOs is given. Then, the LMS-based learning algorithm for the proposed nonlinear adaptive filter is presented. Finally, the rate-dependent hysteresis modeling process is described completely.

### 3.1. GPOs-Based Adaptive Filter

**Figure 5:**
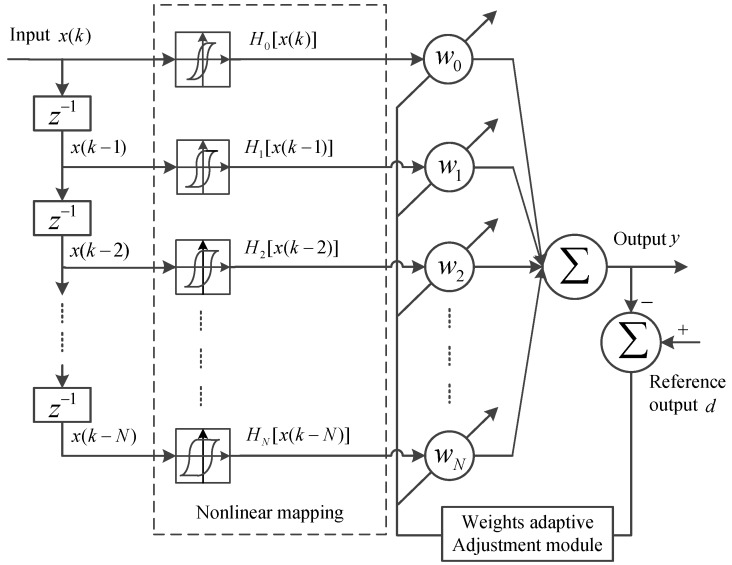
The structure of a generalized play operator (GPO)-based adaptive filter.

The structure of an *N*-th-order GPO-based nonlinear filter is shown in Figure 5. $x\left(k\right)={\left[x\left(k\right)\;x\left(k-1\right)\;\ldots \;x\left(k-N\right)\right]}^{T}$ is the input vector representing a tapped-delay line. GPOs with different thresholds are used to construct a single-layer nonlinear network in the filter. $H\left[x\left(k\right)\right]\;=\;{\left[H_{0}\left[x\left(k\right)\right]\;H_{1}\left[x\left(k-1\right)\right]\;\ldots \;H_{N}\left[x\left(k-N\right)\right]\right]}^{T}$ is the output vector of the GPOs. Based on Equations (3) and (4), the GPOs can be rewritten as:

(12) $$H_{i}\left[x\left(k-i\right)\right]=\left\{\begin{array}{cc} \gamma_{r}\left(x\left(k-i\right)\right)-r_{i}; & x\left(k-i\right)>x\left(k-i-1\right)\;\text{and}\;\gamma_{r}\left(x\left(k-i\right)\right)-r_{i}>H_{i}\left[x\left(k-i-1\right)\right]\\ \gamma_{l}\left(x\left(k-i\right)\right)+r_{i}; & x\left(k-i\right)<x\left(k-i-1\right)\;\text{and}\;\gamma_{l}\left(x\left(k-i\right)\right)+r_{i}<H_{i}\left[x\left(k-i-1\right)\right]\\ H_{i}\left[x\left(k-i-1\right)\right]; & \;\text{otherwise}\; \end{array}\right.$$

The output of the filter can be given as:

(13) $$y\left(k\right)=\sum_{i=0}^{N}w_{i}\left(k-i\right)H_{i}\left[x\left(k-i\right)\right]=w^{T}\left(k\right)H\left[x\left(k\right)\right]$$

where $w\left(k\right)={\left[w_{0}\left(k\right)\;w_{1}\left(k-1\right)\;\ldots \;w_{N}\left(k-N\right)\right]}^{T}$ is the weight vector.

The envelop functions $\gamma_{r}$ and $\gamma_{l}$ in the GPOs depend on the hysteresis characterization of the plant and should be determined based on prior knowledge of the hysteresis modeling plant. In this paper, a systematic identification method for envelop functions used in the filter is presented. Under a quasi-static input signal, which actuates the plant `infinitely slowly’, the output of the GPO-based nonlinear filter can be approximated as:

(14) $$y\left(k\right)=\sum_{i=0}^{N}w_{i}H_{i}\left[x\left(k-i\right)\right]\approx \sum_{i=0}^{N}w_{i}H_{i}\left[x\left(k\right)\right]$$

It can be seen from Equation (14) that under quasi-static input, the GPO-based nonlinear filter can be approximated as a GPI model. Hence, the parameters in the GPOs can be obtained by the GPI model parameter-identification method mentioned in Section 2 using a sufficiently slow actuation signal. An algorithm for the identification of the GPOs in the filter is given as the following:

**Algorithm 1. GPOs algorithm.**

- Step 1. A sufficiently slow input signal v(l),l=0,…,n is generated and applied to the unknown model plant; the output $y_{m}\left(l\right),\;l=0,\ldots ,n$ is measured.
- Step 2. The number of the GPOs in the GPI model is set to the same as the order of the filter *N*.
- Step 3. Initialize the parameters $a_{j},\;j=0,\ldots ,7$ of the envelop function and the weights $w_{r_{i}},i=0,\ldots ,N$.
- Step 4. Calculate the thresholds $r_{i}$ using Equation (10).
- Step 5. Calculate the output of the GPI model $y_{r}\left(k\right)$ using Equation (9).
- Step 6. Determine the parameters $a_{j},\;j=0,\ldots ,7$ and $w_{r_{i}}$, i=0,…,N by minimization of sum-squared-error *J* described by Equation (11).
- Step 7. Calculate the sum-squared-error *J*; if *J* is less than tolerable error *ε*, then end the algorithm; else return to Step 4.

### 3.2. GPO LMS Algorithm

In this section, an LMS-based algorithm is presented for the proposed GPO-based nonlinear filter. This choice can reduce computational complexity, which is attractive for online implementation.

From Equation (13), it is observed that the output of the filter is composed of a linear combination of GPO output signals. It has the same form as the classical linear adaptive filter, except for the form of the input vector. Hence, the LMS algorithm could be used to learn the coefficients in the filter. Most of the analyses and algorithms presented for linear LMS apply equally to the GPO-based filter.

The standard approach for deriving the LMS algorithm is to use an estimate of the mean-square-error (MSE), defined as:

(15) $$E\left[e^{2}\left(k\right)\right]=E\left[d^{2}\left(k\right)-2d\left(k\right)y\left(k\right)+y^{2}\left(k\right)\right]$$

where x(k), y(k) and d(k) are the input signal, the output signal and the reference signal, respectively, and e(k) is the error signal. The instantaneous square error is given by:

(16) $$e^{2}\left(k\right)=d^{2}\left(k\right)-2d\left(k\right)y\left(k\right)+y^{2}\left(k\right)$$

Substituting Equation (13) into Equation (16), the estimate of the MSE objective function can be rewritten as:

(17) $$e^{2}\left(k\right)=d^{2}\left(k\right)-2d\left(k\right)w^{T}\left(k\right)H\left(k\right)+w^{T}\left(k\right)H\left(k\right)H^{T}\left(k\right)w\left(k\right)$$

An LMS-based algorithm can be used to minimize the objective function as follows:

(18) $$\begin{array}{cc} w(k+1) & =w\left(k\right)-\mu {\hat{\nabla }}_{w}\left(k\right)\\ & =w\left(k\right)-2\mu e\left(k\right)\frac{\partial e(k)}{\partial w(k)}\\ & =w(k)+2\mu e(k)H(k) \end{array}$$

for k=0,1,2,…, where ${\hat{\nabla }}_{w}\left(k\right)$ represents an estimate of the gradient vector of the objective function with respect to the filter coefficients, and *μ* is the convergence factor, which controls stability and the convergence speed.

In order to guarantee convergence of the coefficients in the mean, the convergence factor of the GPO-based LMS algorithm must be chosen in the range:

(19) $$0<\mu <\frac{1}{\mathrm{tr}[R]}<\frac{1}{\lambda_{\max}}$$

where $\lambda_{\max}$ is the largest eigenvalue of the input signal vector auto-correlation matrix $R=E[H\left(k\right)H^{T}\left(k\right)]$. The convergence speed of the GPO-based LMS is dependent on the eigenvalue spread of the auto-correlation matrix R.

### 3.3. The Process of Modeling

The modeling steps using the GPO-based adaptive filter for the rate-dependent hysteretic system are given as follows:

(1) Determine the order of the filter *N*. For the unknown model plant, Algorithm 1 is used to identify the envelop function and the threshold values of the GPOs in the filters.
(2) Initialize the weight vector w(0). Determine the convergence factor *μ* based on the auto-correlation matrix R.
(3) Construct the adaptive filter model system. Connect input signal x(k) with the input port of the model plant and the GPO-based adaptive filter. Then, connect the output signal d(k) of the model plant with the output y(k) of the GPO-based adaptive filter using a sum to calculate the error.
(4) Calculate the error signal e(k)=d(k)−y(k). Update the weight vector of the GPO-based adaptive filter through Equation (18).
(5) Provide the next input signal and return to Step 3. Repeat the process until all input signals have been given.

## 4. Model Validation and Experimental Results

**Figure 6:**
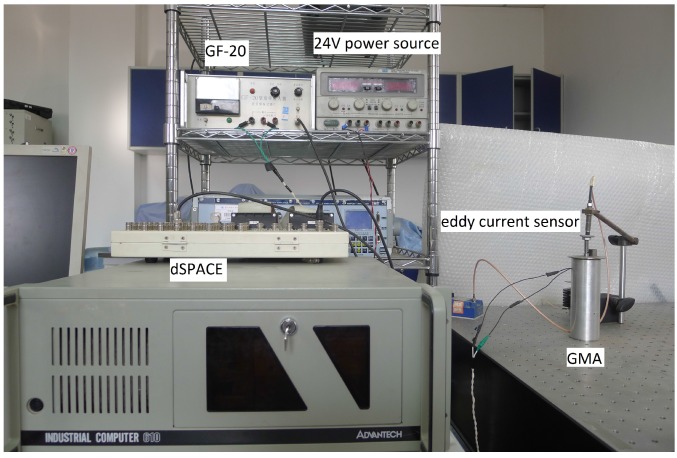
Experimental equipment.

The experimental device was constructed to identify the rate-dependent hysteresis of a GMA system, as shown in Figure 6. The GMA, with a stroke of ±30 μm, was manufactured by Beihang University. The D/A converter transformed the control signal and sent it from the computer to the GMA by means of a current mode power amplifier (GF-20). The displacement was measured by an eddy current sensor with a 8 mV/μm resolution and was transformed via the A/D converter, provided to a dSPACE controller board (DS1103) and recorded by a computer. The sampling frequency was set to 10 kHz.

A low-frequency (1 Hz) sinusoidal input signal was employed to actuate the GMA, and the envelop functions in the filter were identified as follows:

$$\begin{array}{ccc} \gamma_{l} & = & 1.2878\tanh(0.36u(t)+0.6025)-0.6671\\ \gamma_{r} & = & 0.3223\tanh(1.2233u(t)+0.1104)-0.0248 \end{array}$$

We made a comparison of four adaptive filter modeling methods to demonstrate the validation of the proposed modeling method: a GPO-based adaptive filter, a backlash adaptive filter, a second-order series Volterra adaptive filter and a linear adaptive filter. In order to compare the modeling accuracy of the filters, the four adaptive filters should have the same filter order of *N*, and the same algorithms should be used to adjust the coefficient vectors in the various filters.

**Figure 7:**
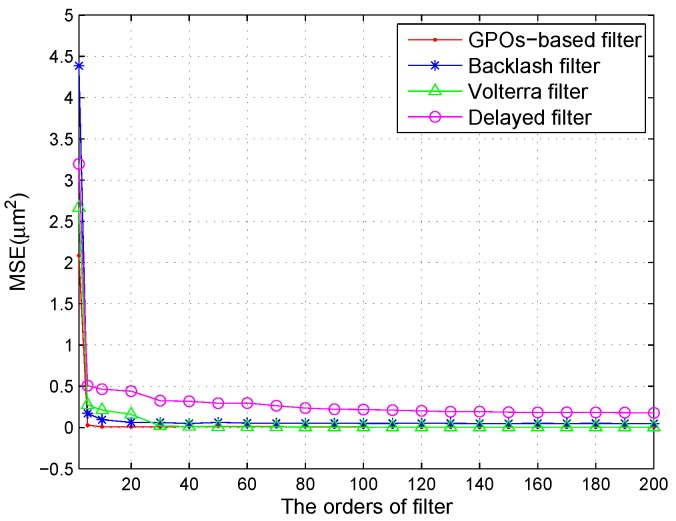
Relationship between MSE and the order of the filters.

Figure 7 illustrates the relationships between MSEs and the order of the filters. The GMA system was actuated by a sinusoidal signal at a frequency of 50 Hz. From Figure 7, it is clear that, for the four adaptive filters, the MSEs decrease sharply when the order of the filters is below 10. When the order of the filters continues to increase, the rate of change of the modeling error is very slow. The length of the coefficient vector of the Volterra filter of the second-order series and of the *N*-th-order was $N+1+{\left(N+1\right)}^{2}$, while those of the other filters of the *N*-th-order were N+1. Considering the accuracy and hardware implementation, the order of the filters was set at 30.

LMS-based algorithms were used as learning algorithms for the four adaptive filters. The convergence factor *μ* governs the convergence speed and the stability of the filter. Many experiments have been done to choose an appropriate *μ* for each filter, in order to achieve optimal modeling performance of the filters. It should be noted that there are different convergence factors for the first-order and second-order terms of the LMS Volterra filters.

Three kinds of speed input signals, including discrete frequency sinusoid signals, chirp signals and sums of sinusoid signals, were used as speed inputs to determine whether the modeling methods can capture the rate-dependent hysteresis characterizations. In order to compare modeling performances, mean square errors (MSEs) and relative errors (REs) of all four adaptive filters under the various excitation signals are shown in Table 1, Table 2 and Table 3.

**Table 1:** Modeling errors for filters under discrete frequency sinusoid signals.

Input		GPOs-Based	Backlash	Volterra	Delayed
Signal		Filter	Filter	Filter	Filter
10 Hz sine wave	MSE (μ$m^{2}$)	0.0094	0.4248	1.1847	0.2880
	RE	0.0106	0.0711	0.1187	0.0585
20 Hz sine wave	MSE (μ$m^{2}$)	0.0095	0.3533	0.5211	0.2882
	RE	0.0108	0.0658	0.0800	0.0594
40 Hz sine wave	MSE (μ$m^{2}$)	0.0098	0.3226	0.2256	0.3560
	RE	0.0114	0.0655	0.0548	0.0688
60 Hz sine wave	MSE (μ$m^{2}$)	0.0103	0.1811	0.1310	0.2450
	RE	0.0131	0.0549	0.0466	0.0638
80 Hz sine wave	MSE (μ$m^{2}$)	0.0122	0.1857	0.1192	0.3258
	RE	0.0134	0.0523	0.0417	0.0690
100 Hz sine wave	MSE (μ$m^{2}$)	0.0119	0.1621	0.0858	0.3168
	RE	0.0141	0.0520	0.0378	0.0726
120 Hz sine wave	MSE (μ$m^{2}$)	0.0123	0.1355	0.0476	0.2828
	RE	0.0151	0.0500	0.0296	0.0723
150 Hz sine wave	MSE (μ$m^{2}$)	0.0157	0.1163	0.0224	0.2514
	RE	0.0183	0.0498	0.0218	0.0732
200 Hz sine wave	MSE (μ$m^{2}$)	0.0221	0.0896	0.0172	0.2071
	RE	0.0246	0.0495	0.0217	0.0736

**Figure 8:**
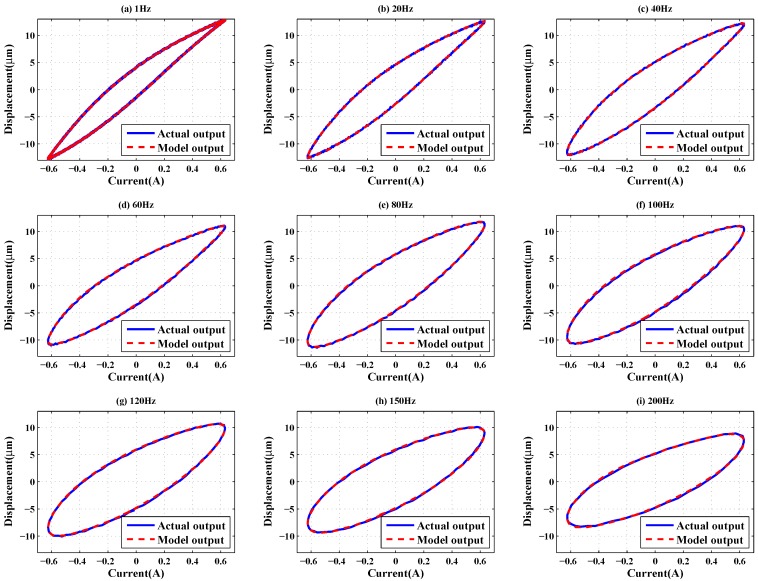
Model validation for single frequency input signals.

First, some discrete frequency sinusoidal signals with amplitudes of 0.632 A were used as inputs (1 Hz, 20 Hz, 40 Hz, 60 Hz, 80 Hz, 100 Hz, 120 Hz, 150 Hz and 200 Hz). Figure 8 gives the modeling results for the GPO-based adaptive filter method. It can be seen from Figure 8 that only major loops of the GMA are actuated by the discrete frequency sinusoidal signals, and the peak-peak displacements change with increasing frequency, owing to the rate-dependent effects of the GMA. From Table 1, it is clear that, under the discrete frequency sinusoidal input signals, the proposed GPO-based adaptive filter has better modeling performance than the other adaptive filters. Especially in the low-frequency range, the modeling errors of the GPO-based adaptive filter are significantly smaller than those of the others. This is mainly because GPOs are used in the proposed adaptive filter, and their parameters are identified through using low-frequency data. The modeling errors of the GPO-based filter increase with increasing frequency. It should be noted that, when the frequency is below 20 Hz, the second-order Volterra adaptive filter gives the worst approximation, and the modeling errors of it sharply decrease with increasing frequency.

**Figure 9:**
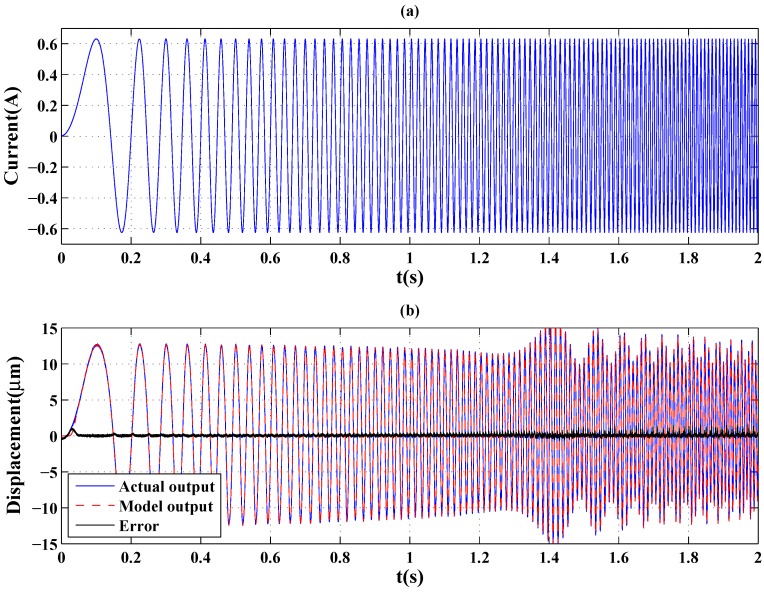
(**a**) A chirp signal in which the frequency increases linearly with time from 1 Hz to 100 Hz; (**b**) model output displacement of the GPO-based adaptive filter and error.

A chirp signal with an amplitude 0.632 A, in which the frequency increased linearly with time from 1 Hz to 100 Hz as shown in Figure 9a, was then used to actuate the GMA system. The modeling result of the GPO-based adaptive filter is shown in Figure 9b. From Figure 9, it is clear that, when the time is less than 1.28 s (a frequency of about 63.4 Hz), the peak-peak displacement of the GMA decreased with increasing frequency. A remarkable increase of the peak-peak displacement is observed near 1.41 s (a frequency of about 69.7 Hz) from Figure 9, which was due to the first-order resonance frequency of 69.7 Hz of the GMA system and which causes oscillation of the displacement when the frequency continues to increase. The coupling of the hysteresis effects and resonance behavior of the GMA make the rate-dependent hysteresis modeling more difficult. From Table 2, it is clear that, under the chirp signal, the modeling errors of the GPO-based adaptive filter are smaller than those of the other adaptive filters.

**Table 2:** Modeling errors under a chirp signal.

Input		GPO-Based	Backlash	Volterra	Delayed
Signal		Filter	Filter	B	Filter
Chirp signal	MSE (μ$m^{2}$)	0.0646	0.4356	0.4882	0.6300
Figure 9a	RE	0.0296	0.0769	0.0814	0.0924

Finally, the proposed modeling method was validated by using the sums of the sinusoidal signals to actuate the GMA system. Two signals were generated as:

$$\begin{array}{ccc} u & = & 0.1\sin(2\pi 10t)+0.2\sin(2\pi 30t)+0.3\sin(2\pi 50t)\\ u & = & 0.12\sin(2\pi 5t)+0.12\sin(2\pi 25t)+0.12\sin(2\pi 50t)+0.12\sin(2\pi 75t)+0.12\sin(2\pi 100t) \end{array}$$

**Figure 10:**
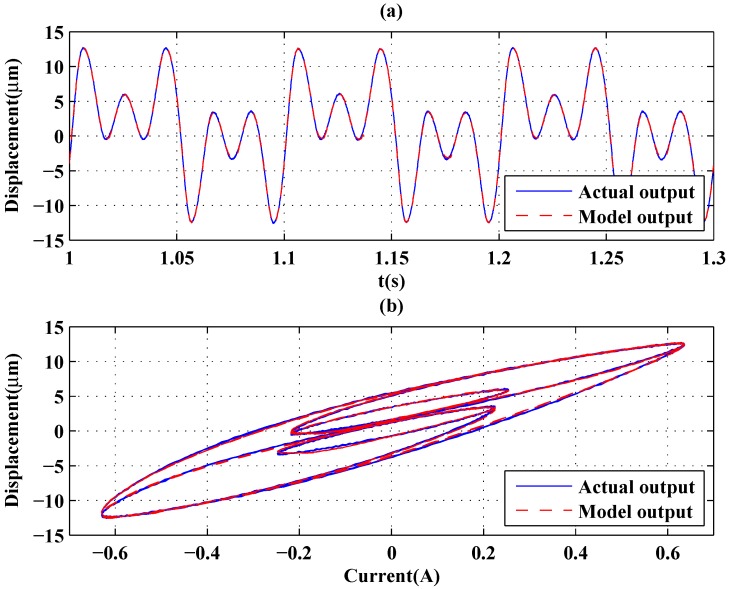
Modeling: output of the model and actuator (sum of sinusoids at 10, 30 and 50 Hz). (**a**) Outputs of the model and actuator; (**b**) hysteresis curves of the model and actuator.

**Figure 11:**
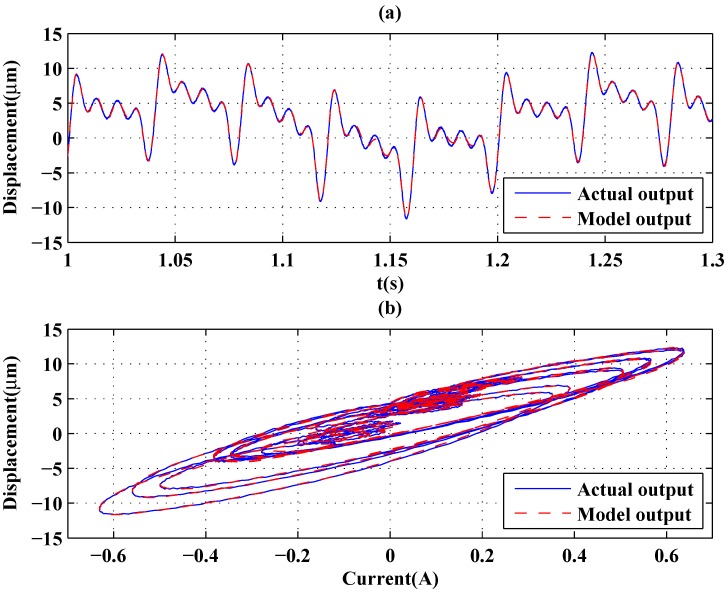
Modeling: output of the model and actuator (sum of sinusoids at 5, 25, 50, 75 and 100 Hz). (**a**) Outputs of the model and actuator; (**b**) hysteresis curves of the model and actuator.

**Figure 12:**
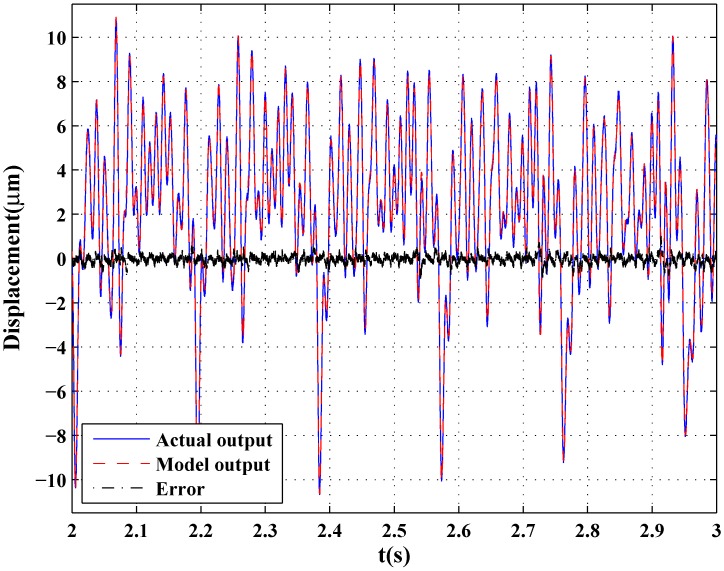
Modeling results under a sum of sinusoids containing 0–100 Hz frequency information characteristics.

Figure 10 and Figure 11 show the two input signals and modeling results of the GPO-based adaptive filter, respectively. Furthermore, a more complicated signal containing 0–100 Hz frequency information characteristics was generated using the idinput command in MATLAB and was applied as speed input in which the frequency band expressed in fractions of the Nyquist frequency was set as [0.0 0.02], and the level was set at 0.5. Figure 12 shows the modeling result of the GPO-based filter under this signal. By using the sums of the sinusoidal signals as inputs, the complicated hysteresis characteristics of nonlocal memory effects were revealed, as shown in Figure 10, Figure 11 and Figure 12. From Table 3, it can be seen that the proposed GPO-based adaptive filter has a remarkable ability to model complicated rate-dependent hysteresis nonlinearity compared to the other adaptive filters. The backlash filter also shows better modeling performance than the second-order Volterra adaptive filter and linear delay adaptive filter, owing to their use of backlash operators.

**Table 3:** Modeling results under the sums of sinusoidal signals.

Input		GPOs-Based	Backlash	Volterra	Delayed
Signal		Filter	Filter	Filter	Filter
Sum of sinusoid signal	MSE (μ$m^{2}$)	0.0186	0.0430	1.6099	1.6897
(10, 30, 50 Hz), Figure 10	RE	0.0205	0.0312	0.1910	0.1954
Sum of sinusoid signal	MSE (μ$m^{2}$)	0.0351	0.0882	1.8882	2.2469
(5, 25, 50, 75, 100 Hz), Figure 11	RE	0.0370	0.0608	0.2907	0.3069
Sum of sinusoid signal	MSE (μ$m^{2}$)	0.0425	0.0939	1.6865	1.4340
Figure 12	RE	0.0470	0.0699	0.2964	0.2733

Moreover, we compare the proposed rate-dependent adaptive filter modeling method with a phenomenological operator-based model, for example the rate-dependent generalized Prandtl-Ishlinskii (RDGPI) model [16,32]. The RDGPI model describes the rate-dependent hysteresis behaviors by extending the rate-independent threshold vector and weight vector to the rate-dependent ones. The inversion of the RDGPI can be formulated analytically, which is attractive for the inverse compensation design. A discrete RDGPI model [32] is used to model the rate-dependent hysteresis nonlinearities of the GMA system. The parameters of the RDGPI model are obtained by minimization of the error function over 10–100 Hz range of input frequencies. The detailed identification method can be found in [32], which results in $\alpha_{1}=8.3721$, $\alpha_{2}=0.0785$, $\beta_{1}=1.002$, $\beta_{2}=1.2443$, $\lambda_{1}=0.2494\;\times$ 10${}^{-5}$, $\lambda_{2}=0.94\;\times$ 10${}^{-2}$, c=1.1867, ρ=3.3478, ξ=7.0270, τ=0.1910 and μ=−0.1284. Table 4 gives the modeling performances of the RDGPI under the different excitation inputs. It can be seen from Table 4 that the proposed GPO-based adaptive filter has a remarkable superiority in modeling the complicated dynamic response of the smart structure owing to its adaptive filter structure.

**Table 4:** Modeling results of RDGPI under different excitation signals.

Input Signal		RDGPI Model	GPO-Based Filter
10 Hz sine wave	MSE(μ$m^{2}$)	1.2298	0.0094
	RE	0.1172	0.0106
20 Hz sine wave	MSE(μ$m^{2}$)	0.8262	0.0095
	RE	0.0974	0.0108
40 Hz sine wave	MSE(μ$m^{2}$)	0.7155	0.0098
	RE	0.0945	0.0114
60 Hz sine wave	MSE(μ$m^{2}$)	0.5148	0.0103
	RE	0.0835	0.0131
80 Hz sine wave	MSE(μ$m^{2}$)	0.9183	0.0122
	RE	0.1084	0.0134
100 Hz sine wave	MSE(μ$m^{2}$)	0.6111	0.0119
	RE	0.1	0.0141
Chirp signal	MSE(μ$m^{2}$)	2.6650	0.0646
	RE	0.2225	0.0296
Sum of sinusoid signal	MSE(μ$m^{2}$)	2.7005	0.0186
(10, 30, 50 Hz)	RE	0.2474	0.0205
Sum of sinusoid signal	MSE(μ$m^{2}$)	3.3587	0.0351
(5, 25, 50, 75, 100 Hz)	RE	0.3850	0.0370
Sum of sinusoid signal	MSE(μ$m^{2}$)	3.7751	0.0425
Figure 9a	RE	0.3446	0.0470

## 5. Conclusions

A novel nonlinear adaptive filter was proposed for rate-dependent hysteresis modeling, where the tapped-delay line was used as the input signal vector and was mapped into another signal vector through a single-layer network containing GPOs with various thresholds. An LMS-based algorithm was used to adjust the coefficient vector in the adaptive filter. A GMA system was used as a model plant, and three kinds of speed signals were used to actuate the strongly rate-dependent hysteresis characteristics of the GMA. A comparison of various modeling methods was made to demonstrate the validation of the proposed adaptive filter. Experimental results showed the effectiveness of the proposed rate-dependent hysteresis modeling method.
